# 936. Antimicrobial Stewardship Assessments in De-labeling Beta-lactam Allergies

**DOI:** 10.1093/ofid/ofac492.780

**Published:** 2022-12-15

**Authors:** Nicole Fahmy, Faiza Morado, Neha Nanda

**Affiliations:** Keck Medical Center of USC, Los Angeles, California; Keck Medical Center of USC, Los Angeles, California; USC, los angeles, California

## Abstract

**Background:**

Unnecessary use of broad-spectrum antibiotics due to false antibiotic allergy labels leads to an increase in drug resistant and health care associated infections. Therefore, allergy assessments to de-label patients not truly allergic is an important antimicrobial stewardship (AMS) tool. The purpose of this study was to assess the effectiveness of a pharmacy-led questionnaire and AMS rounds in improving the accuracy of beta-lactam allergy labels (BLA).

**Methods:**

AMS piloted BLA de-labeling rounds three times a week from November 1, 2021 to April 1, 2022. Rounds consisted of an infectious disease physician and an AMS pharmacist that reviewed allergy questionnaires conducted by trained pharmacy personnel. The questionnaire was provided to patients 18 years or older with a documented BLA and an admission stay of two or more nights. Patients were excluded if unable to participate or did not have a representative available for interview. The primary outcomes evaluated were: (i) direct de-labelling (ii) future de-labeling upon patient consent (iii) allergy profile changes. The secondary outcomes were potential days of therapy (DOTs) saved for anti-MRSA agents (vancomycin, linezolid, clindamycin) and fluoroquinolones (ciprofloxacin, levofloxacin) and PEN-FAST scores for those with a penicillin-specific allergy.

**Results:**

93 of 96 patients interviewed (96.9%) had their allergy profile updated to include type, severity, or timing of reaction. 82 of 96 patients (85.4%) were classified as low-risk allergies in which 11 patients (13.4%) were successfully de-labeled and 18 patients (22.0%) were eligible for de-labeling upon patient consent. The remaining patients were eligible for further interrogation with an oral challenge (Figure 1). Potential DOTs saved were 131 and 67 days for anti-MRSA agents and fluoroquinolones, respectively. Average PEN-FAST scores for those de-labeled and eligible for oral challenge was 0.5 (SD 0.85) and 1.08 (SD 1.20), respectively.

**Figure:**
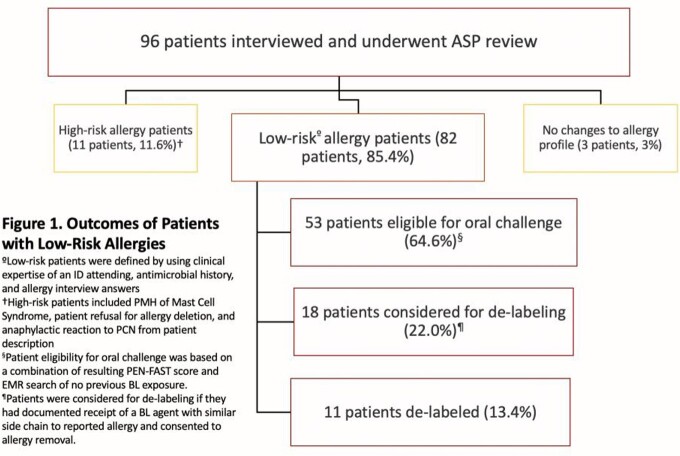

**Conclusion:**

AMS BLA assessments can decrease the prevalence of false BLA labels and prescribing of unnecessary broad-spectrum agents. PEN-FAST scores less than 2 suggests that allergy assessments help identify patients with low-risk penicillin allergies.

**Disclosures:**

**All Authors**: No reported disclosures.

